# Current Trends in Organ Preservation Solutions for Pancreas Transplantation: A Single-Center Retrospective Study

**DOI:** 10.3389/ti.2022.10419

**Published:** 2022-03-28

**Authors:** Joana Ferrer-Fàbrega, Emma Folch-Puy, Juan José Lozano, Pedro Ventura-Aguiar, Gabriel Cárdenas, David Paredes, Ángeles García-Criado, Josep Antoni Bombí, Rocío García-Pérez, Miguel Ángel López-Boado, Ramón Rull, Enric Esmatjes, Maria José Ricart, Fritz Diekmann, Constantino Fondevila, Laureano Fernández-Cruz, Josep Fuster, Juan Carlos García-Valdecasas

**Affiliations:** ^1^ Hepatobiliopancreatic Surgery and Liver and Pancreatic Transplantation Unit, Clinic Institute of Digestive and Metabolic Diseases (ICMDiM), Hospital Clínic, University of Barcelona, Barcelona, Spain; ^2^ August Pi i Sunyer Biomedical Research Institute (IDIBAPS), Barcelona, Spain; ^3^ Experimental Pathology Department, Institut d’Investigacions Biomèdiques de Barcelona (IIBB), Consejo Superior de Investigaciones Científicas (CSIC), Barcelona, Spain; ^4^ Bioinformatics Platform, Network for Biomedical Research in Hepatic and Digestive Diseases (CIBERehd), Barcelona, Spain; ^5^ Network for Biomedical Research in Hepatic and Digestive Diseases (CIBERehd), Barcelona, Spain; ^6^ Renal Transplant Unit, Nephrology and Kidney Transplant Department, Hospital Clínic, University of Barcelona, Barcelona, Spain; ^7^ Donation and Transplant Coordination Unit, Hospital Clínic, University of Barcelona, Barcelona, Spain; ^8^ Department of Radiology, Hospital Clínic, University of Barcelona, Barcelona, Spain; ^9^ Department of Pathology, Hospital Clínic, University of Barcelona, Barcelona, Spain; ^10^ Diabetes Unit, Department of Endocrinology and Nutrition, Hospital Clínic, University of Barcelona, Barcelona, Spain

**Keywords:** pancreas transplantation, graft survival, preservation solution, ischemia-reperfusion, pancreatitis, postoperative outcomes

## Abstract

Due to the high vulnerability of the pancreas to ischemia-reperfusion injury, choices regarding preservation solution markedly affect pancreas transplant success. A retrospective single-center analysis of 380 pancreas transplants (2000–2019) was performed to correlate current preservation solutions with transplant outcomes. Early graft failure requiring transplantectomy within 30 days post-transplant occurred in 7.5% for University of Wisconsin (UW) group (*n* = 267), 10.8% of Celsior (CS) group (*n* = 83), 28.5% of Histidine-Tryptophan-Ketoglutarate (HTK) group (*n* = 7), and none for Institut Georges Lopez-1 (IGL-1) group (*n* = 23). The most common causes of technical failures in this cohort included abdominal hemorrhage (8.4%); graft pancreatitis (3.7%); fluid collections (2.6%); intestinal complications (6.6%); and vascular thrombosis (20.5%). Although IGL-1 solution provided lower surgical complication rates, no significant differences were found between studied groups. Nevertheless, HTK solution was associated with elevated pancreatitis rates. The best graft survival was achieved at 1 year using UW and IGL-1, and at 3 and 5 years using IGL-1 (*p* = 0.017). There were no significant differences in patient survival after a median follow-up of 118.4 months. In this setting therefore, IGL-1 solution appears promising for perfusion and organ preservation in clinical pancreas transplantation, compared to other commonly used solutions.

## GRAPHICAL ABSTRACT

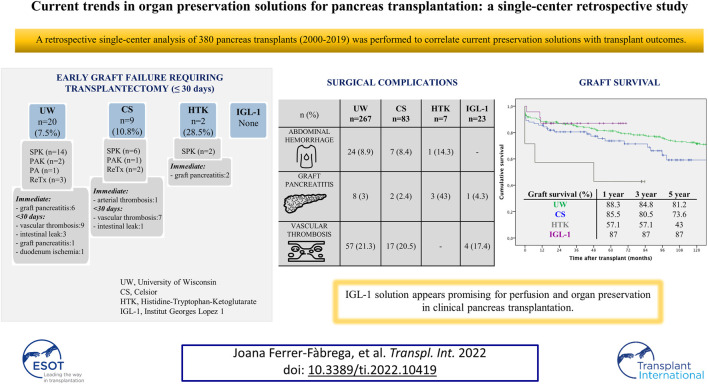

## Introduction

For patients with diabetes mellitus (DM) type 1, pancreas transplantation (PTx) is the only therapeutic option capable of normalizing blood glucose and minimizing secondary complications of diabetes, resulting in an increase in the survival and an improved quality of life (1). According to data from the International Pancreas Transplant Registry, more than 56,000 PTx’s were carried out worldwide between the first operation in the 1960s and 2017 (2). In Spain, with 12 accredited centers, 2,006 PTx’s have been performed since the program started in 1983 (3-5).

The maintenance of organ viability from donation to transplantation is a decisive factor for the adequate function and survival of the graft, especially in organs such as the pancreas, which is highly susceptible to ischemic damage. Preservation has become a key challenge due to the increasing use of marginal donors, in whom the functionality of the organ is most affected (6,7).

In this scenario, four preservation solutions are currently in use for pancreas transplantation. University of Wisconsin (UW) solution has been considered for organ perfusion in abdominal organ transplantation since the late 80s (8). It features a potassium concentration that mimics the intracellular medium and uses hydroxyethyl starch (HES) as the oncotic agent. In contrast, Histidine-Tryptophan-Ketoglutarate (HTK) and Celsior (CS) solutions, which were originally designed for cardiac graft protection, have the advantage of a much lower viscosity, providing more rapid cooling and better washout during organ procurement. Meanwhile, Institut Georges Lopez 1 (IGL-1) preservation solution was introduced in the early 2000s for the maintenance of abdominal organs and, although clinical experience in PTx with this solution is limited, initial results have been promising (9). Its composition resembles that of UW, with inversed potassium/sodium contents and replaced HES [with a tendency to induce red blood cell aggregation (10)] with 35-kDa molecular-weight polyethylene glycol (PEG35), a neutral, water-soluble, non-toxic polymer that acts like a colloid (11).

At present there is no universal consensus regarding the optimal preservation solution in the setting of PTx albeit UW solution continues to be recognized as the “gold standard” (12). Considering that early technical failure remains the Achilles’ heel of pancreas transplantation, there is a growing need within the scientific community for new solutions with superior preservation properties and reduced side effects.

In recent years, the Pancreatic Transplant Unit at Hospital Clínic of Barcelona has routinely used IGL-1 as a preservation solution for PTx from its own donors. The aim of this study was to compare the effectiveness of the four currently in-use preservation solutions on the outcome of PTx regarding early pancreatic graft function as well as long-term patient and graft survival. Secondly, postoperative surgical complications were also evaluated, as well as their relation with ischemia-reperfusion injury.

## Material and Methods

### Study Design

Five hundred ninety-one consecutive pancreas transplants were performed at the Hospital Clínic of Barcelona from 1983 through to the end of 2019. A prospectively assembled database of all pancreas transplants from January 2000 to March 2019 was reviewed, i.e. since surgical technique and immunosuppression strategies were standardized. The patient cohort included 380 patients who underwent PTx: 312 (82.1%) simultaneous pancreas-kidney (SPK); 27 (7.1%) pancreas after kidney (PAK) and 3 (0.8%) pancreas transplant alone (PTA). In addition, 38 (10%) patients received a pancreas retransplantation. Data from this cohort were stratified into four groups according to the organ preservation solution employed (UW, CS, HTK and IGL-1). UW and CS were used throughout the whole period of analysis, HTK from January to December 2013 while IGL-1 has been in use from 2014 to the present.

This study protocol was approved by our institutional review board (HCB/2020/0499) and complied with the ethical standards of the Helsinki Declaration of 1975.

### Donor Characteristics

Graft pancreas acceptance criteria was performed based on the consensus document of the National Transplant Organization described in 2005 and updated in 2018 (13). Donor analyzed characteristics included: age; gender; cause of death; body mass index (BMI); cold ischemia time (CIT); pre-procurement pancreas suitability score (PPASS); perfusion volume, and amylase/lipase levels.

During organ procurement, both abdominal aorta and portal vein cannulation (dual perfusion) were used to perfuse the organs (perfusion time 8–10 min). The perfusion volume differed depending on the surgeon criteria to obtain a clear effluent via vena cava. The standard, whole-pancreas graft included the entire pancreas and a duodenal segment.

### Recipient Characteristics

The indications for PTx were patients with DM who met the inclusion criteria according to the protocol established in our institution (14). Venous systemic drainage was performed between graft portal vein and recipient vena cava or right iliac vein. Arterial supply for the pancreatic graft was done through the anastomosis of the recipient right iliac primitive artery to the graft superior mesenteric artery or the common iliac graft artery, depending on the backtable reconstruction (15). For exocrine secretion, enteric drainage was created “side-to-side”, either by duodeno-jejunostomy (from January 2000 to April 2016) or duodeno-duodenostomy anastomosis (from May 2016 to March 2019).

The demographic recipient factors included age; gender; BMI; DM type-1; time of DM evolution (DM *vintage*), and type and duration of dialysis (Dialysis *vintage*). In addition, surgical complications were defined according to the modified Clavien Dindo classification (16) as any postoperative event related to the procedure within the 90 days following the transplant. Postoperative hemorrhage was classified according to the definition of the International Study Group for Pancreatic Surgery (ISGPS) (17). As there was a lack of consensus regarding a clear definition of graft pancreatitis, it was considered the case when it was readily apparent that it had arisen intraoperatively from ischemia-reperfusion injury and its related-complications such as pancreatic abscesses, and peripancreatic fluid collections. Other entities were also considered such as sterile or infected abdominal fluid collections either diagnosed by ultrasound/abdominal computed tomography or evidenced by clinical symptoms. Intestinal complications included duodenum-related leaks and small-bowel obstruction.

Early pancreatic graft function was evaluated both by biochemical parameters (peak serum amylase and lipase levels in the first 48 h together with insulin requirements) and by clinical outcomes, including the need of transplantectomy within 30 days of transplantation.

### Immunosuppression

Routine immunosuppression in SPK and PAK consisted of different regimens administered following the institutional protocol, which varied according to the date of transplant including monoclonal antibody (OKT3), anti-interleukin-2 monoclonal antibody (basiliximab), rabbit anti-human lymphocytes polyclonal antibodies (thymoglobulin) among others, as standard induction therapy. Maintenance immunosuppression was based on triple therapy with calcineurin inhibitor (cyclosporine A until 2005 vs. tacrolimus introduced in the late 90s), mycophenolate and steroids.

### Anticoagulant Therapy and Antibiotic Prophylaxis

Our standard anticoagulation protocol included enoxaparin 20 mg every 12 h, starting 8-h post-surgery and maintained until patient discharge (in the absence of thrombotic/hemorrhagic complications), and aspirin 50 mg/d starting at 12-h post-surgery until discharge (100 mg/d).

Vancomycin plus third-generation cephalosporin (from 2000 to 2014) or ertapenem (from 2015 to 2019) were used as antibiotic prophylaxis in the perioperative period. Fungal prophylaxis with fluconazole was universally used in all recipients. Cytomegalovirus prophylaxis was provided by ganciclovir or valganciclovir, depending on glomerular filtration rates.

### Statistical Analysis

Categorical variables are expressed as frequencies (%), percentages and continuous variables such as median and interquartile range (IQR). Categorical variables were analyzed by use of Fisher’s exact or χ^2^ test. Mann-Whitney U test or the Kruskal Wallis in the case of nonparametric distribution were used for the analysis of continuous variables. Due to the limited number of cases for HTK group, and the resulting bias that may arise in subgroup analysis, we have deemed it appropriate to provide a detailed description of the immediate post-transplant complications instead of including it for comparison with other groups.

The following variables have been included in the univariate and multivariate analysis as potential risk factors for early graft survival: donor demographics (age, gender, cause of death, body mass index, amylase and lipase values, and P-PASS); donor procurement factors (preservation solution, total perfusion volume, cold ischemia time); era of transplant (before and after 2010); recipient demographics (age, gender, body mass index, DM type, DM vintage, dialysis vintage, type of dialysis, transplant type, and induction therapy). Other factors related to surgical management and technique included were the type of arterial reconstruction in the backtable, the type of vascular (arterial and venous) anastomosis and the intestinal anastomosis technique used in the recipient.

Patient and graft survival were assessed using Kaplan–Meier curves and compared with the log-rank test (LR) and Breslow. Numeric covariates were dichotomized by their median. Patient survival was calculated from the time of transplant to death or the end of follow-up. Pancreas graft survival was calculated from the time of transplant until any of the following: the need for graft removal; the return to permanent insulin therapy dependency; retransplant or death/end of follow-up with a functioning graft. *p* values of less than 0.05 were considered statistically significant. Significant covariates were subjected to multivariate cox regression analysis.

Statistical calculations were made using SPSS for Windows software (IBM SPSS Statistics version 20.0, 1989–1995; Chicago, IL) and R statistical software (R Core Team (2017). R: A language and environment for statistical computing. R Foundation for Statistical Computing, Vienna, Austria. URL https://www.R-project.org/).

## Results

### Demographic Profile

A total 380 PTx’s were performed in our center with the use of four different preservation solutions, which differed in terms of their chemical composition (Table 1). Some 267 (70.3%) patients were perfused with UW, 83 (21.8%) with CS, 7 (1.8%) with HTK, and 23 (6.1%) with IGL-1. HTK was introduced in 2013 but was associated with a high and unexpected incidence of graft pancreatitis, prompting us to cease using it and convert to IGL-1.

**TABLE 1:** Components and function of the various preservation solutions compared in the study.

	**UW**	**CS**	**HTK**	**IGL-1**	**Function**
mOsm/L	320	320	310	290	—
Na^+^	30	100	15	120	Maintenance of osmotic balance
K^+^	125	15	10	25	Maintenance of osmotic balance
Cl^−^	—	—	50	—	Maintenance of osmotic balance
Mg^2+^	5	13	4	—	Maintenance of osmotic balance
Ca^2+^	—	0.25	0.015	0.5	Maintenance of osmotic balance
HCO^3-^	5	—	—	—	Buffer
SO^4-^	5	—	—	5	Buffer
PO^4-^	25	—	—	25	Buffer
HES (g/L)	50	—	—	—	Oncotic agent, impermeant
PEG35 (g/L)	—	—	—	1	Oncotic agent, impermeant
Mannitol	—	60	30	—	Impermeant, membrane stabilizer
Lactobionate	100	80	—	100	Impermeant, membrane stabilizer
Raffinose	30	—	—	30	Impermeant
Allopurinol	1	—	—	1	Antioxidant
Histidine	—	30	180	—	Antioxidant, buffer
Tryptophan	—	—	2	—	Antioxidant, membrane stabilizer
Glutathione	3	3	—	3	Antioxidant
Ketoglutarate	—	—	1	—	Energy metabolism substrate
Adenosine	5	—	—	5	Energy metabolism substrate
Glutamate	—	20	—	—	Energy metabolism substrate

Concentrations are expressed in mmol/L, unless otherwise specified.

HES, indicates hydroxyethyl starch; PEG35, polyethylene glycol 35 kDa; UW, University of Wisconsin; CS, Celsior; HTK, Histidine-Tryptophan-Ketoglutarate; IGL-1, Institut Georges Lopez-1.

The four groups had similar characteristics regarding donors as shown in Table 2. HTK and CS groups presented older donor age as compared to IGL-1 and UW (*p* < 0.05). IGL-1 and HTK exhibited shorter CIT (*p* < 0.05), with significantly larger volumes of perfusion solution as compared to CS and UW (*p* < 0.05). The preservation solutions did not differ regarding gender, cause of death, BMI, PPASS and the levels of lipase. Nevertheless, in relation to donor amylase levels, HTK group presented lower values compared to others.

**TABLE 2:** Relationship between preservation solutions and clinicopathological features of donors.

	**Total *n* = 380**	**UW *n* = 267**	**CS *n* = 83**	**HTK *n* = 7**	**IGL-1 *n* = 23**	***P***
Age (years)	32 (21–40)	30 (20–39)	37 (29–45)	43 (33–47)	30 (19–39)	0.803^a^
						0.042^b^
						<0.001^c^
Gender M/F	224 (58.9)/156 (41.1)	164 (61.4)/103 (38.6)	45 (54.2)/38 (45.8)	4 (57.1)/3 (42.9)	11 (47.8)/12 (52.2)	0.266^a^
						0.642^b^
						0.251^c^
Cause of death						
-Trauma	197 (51.8)	153 (57.3)	31 (37.3)	2 (28.6)	11 (47.8)	0.561^a^
-Anoxic damage	21 (5.5)	14 (5.2)	5 (6)	-	2 (8.7)	0.100^b^
-CVA	146 (38.4)	90 (33.7)	44 (53)	4 (57.1)	8 (34.8)	0.012^c^
-Others	16 (4.2)	10 (3.7)	3 (3.6)	1 (14.3)	2 (8.7)	
BMI (kg/m^2^)	23.4 (21.5–25.3)	23.2 (21.3–25.2)	23.4 (22.3 25.5)	24.2 (23.1–27.3)	23.6 (20.8–25.6)	0.839^a^
						0.418^b^
						0.065^c^
Pancreas CIT (hours)	10.1 (8–12)	10 (8–12)	11 (9–12.1)	8.3 (6–10.3)	8.2 (7.1–10.1)	0.001^a^
						<0.001^b^
						0.115^c^
Kidney CIT (hours)	12.3 (10–14.3)	12.3 (10–14.3)	12.8 (10.2–14.7)	10.8 (9.4–14.1)	11.2 (9.9–12.8)	0.262^a^
						0.188^b^
						0.600^c^
PPASS	16 (14–18)	16 (14–18)	17 (14–18)	17 (15–20)	17 (14–18)	0.637^a^
						0.683^b^
						0.043^c^
Perfusion Volume (L)	6.8 (6.0–7.4)	6.5 (6.0–7.0)	6 (5–6.1)	7 (6–7.5)	7.5 (7–8)	0.014^a^
						0.002^b^
						0.099^c^
Amylase (IU/L)	84 (47–164.2)	86 (48–172)	73 (39–146)	51 (39–63)	94 (57–294)	0.629^a^
						0.202^b^
						0.112^c^
Lipase (IU/L)	45 (17–109)	50 (20–126)	22 (11–85.5)	29 (8.2–55.7)	33 (6–79)	0.088^a^
						0.820^b^
						0.091^c^

Continuous variables are expressed as median (interquartile ranges) and categorical variables as frequencies (percentages).

Comparison of the analyzed variables have been made between UW, CS, and IGL-1, groups. For HTK, group only a descriptive analysis is displayed.

aIGL-1, vs. UW; bIGL-1, vs. CS; cUW, vs. CS.

M, indicates male; F, female; CVA, cerebrovascular accident; BMI, body mass index; CIT, cold ischemia time; PPASS, pre-procurement pancreas suitability score; UW, University of Wisconsin; CS, Celsior; HTK, Histidine-Tryptophan-Ketoglutarate; IGL-1, Institut Georges Lopez-1.

Recipient demographics showed no significant differences with respect to gender, BMI, dialysis *vintage*, type of dialysis and type of transplant (Table 3). By contrast, recipients in the IGL-1 group were older compared with UW group (*p* = 0.003) and had the lower proportion of patients with DM I compared to others. Thymoglobulin was the most frequently used drug as induction therapy for CS, HTK and IGL-1 groups.

**TABLE 3:** Relationship between preservation solutions and clinicopathological features of recipients.

	**Total *n* = 380**	**UW *n* = 267**	**CS *n* = 83**	**HTK *n* = 7**	**IGL-1 *n* = 23**	***P***
Age (years)	40 (35–45)	39 (34–44)	42 (37–47)	45 (33–49)	47 (37–53)	0.003^a^
						0.218^b^
						0.001^c^
Gender M/F	240 (63.2)/140 (36.8)	170 (63.7)/97 (36.3)	55 (66.3)/28 (33.7)	3 (42.9)/4 (57.1)	12 (52.2)/11 (47.8)	0.369^a^
						0.231^b^
						0.696^c^
BMI (kg/m^2^)	22.7 (20.9–25.6)	22.4 (20.6–25.5)	23 (20.7–25.7)	22.5 (21.2–26.1)	23.1 (21.8–25.5)	0.198^a^
						0.581^b^
						0.402^c^
DM type						<0.001^a^
-DM I	374 (98.4)	266 (99.6)	80 (96.4)	7 (100)	21 (91.3)	0.017^b^
-Others	6 (1.6)	1 (0.4)	3 (3.6)		2 (8.7)	0.015^c^
DM *vintage* (years)	26 (21–31)	25 (21–30)	28 (22–33.2)	32 (25–34)	27 (21–37)	0.182^a^
						0.904^b^
						0.020^c^
Dialysis *vintage* (months)	26.5 (17.4–36.7)	26 (18–36.7)	26.8 (19–36.1)	47.3 (26.4–52.3)	24 (11.5–35.3)	0.361^a^
						0.322^b^
						0.917^c^
Type of dialysis						
-Hemodialysis	213 (56)	151 (56.6)	36 (43.4)	5 (71.4)	12 (52.2)	0.846^a^
-Peritoneal dialysis	85 (22.4)	63 (23.6)	22 (26.5)	2 (28.6)	5 (21.7)	0.992^b^
-Pre-emptive	30 (7.9)	20 (7.5)	9 (10.8)		2 (8.7)	0.469^c^
-No dialysis	52 (13.7)	33 (12.3)	9 (10.8)		4 (17.4)	
Transplant type						
-SPK	312 (82.1)	224 (83.9)	62 (74.7)	7 (100)	19 (82.6)	0.933^a^
-PAK	27 (7.1)	16 (6)	9 (10.8)	—	2 (8.7)	0.847^b^
-PA	3 (0.8)	2 (0.7)	1 (1.2)	—	—	0.281^c^
-Retransplant	38 (10)	25 (9.4)	11 (13.3)	—	2 (8.7)	
Induction therapy						
-Basiliximab	151 (39.7)	116 (43.5)	32 (38.5)	3 (42.8)	-	<0.001^a,b^
-Thymoglobulin	192 (50.5)	114 (42.7)	51 (61.5)	4 (47.2)	23 (100)	0.001^c^
-Others	37 (9.8)	37 (13.8)	—	—	—	
Graft reconstruction						
-SA-SMA	350 (92.1)	249 (93.3)	72 (86.7)	7 (100)	22 (95.7)	0.823^a^
-“Y” iliac graft	27 (7.1)	15 (5.6)	11 (13.3)	—	1 (4.3)	0.191^b^
-Others	3 (0.8)	3 (1.1)	—	—	—	0.012^c^
Intestinal anastomosis	—	—	—	—	—	
-Duodeno-jejunostomy	337 (88.7)	256 (95.9)	67 (80.7)	7 (100)	7 (30.4)	<0.001^a,b,c^
-Duodeno-duodenostomy	43 (11.3)	11 (4.1)	16 (19.3)	—	16 (69.6)	
Transplant Era						
-2000–2009	226 (59.5)	220 (82.4)	6 (7.2)	—	—	<0.001^a,c^
-2010–2019	154 (40.5)	47 (117.6)	77 (92.7)	7 (100)	23 (100)	0.336^b^

Continuous variables are expressed as median (interquartile ranges) and categorical variables as frequencies (percentages).

Comparison of the analysed variables have been made between UW, CS, and IGL-1, groups. For HTK, group only a descriptive analysis is displayed.

aIGL-1, vs. UW; bIGL-1, vs. CS; cUW, vs. CS.

M, indicates male; F, female; BMI, body mass index; DM, Diabetes Mellitus; SPK, Simultaneous Pancreas-Kidney; PAK, Pancreas After Kidney; PA, Pancreas Transplant Alone; SA-SMA, Splenic Artery - Superior Mesenteric Artery; UW, University of Wisconsin; CS, Celsior; HTK, Histidine-Tryptophan-Ketoglutarate; IGL-1, Institut Georges Lopez-1.

### Surgical Technique

There were, by far, more SPK compared to PAK and PTA in the UW, CS and IGL-1 group (Table 3). Patients transplanted with HTK solution corresponded solely to SPK technique.

For the vascular reconstruction of the pancreatic graft during backtable, arterial anastomosis between the splenic artery and the superior mesenteric artery was performed in the majority of cases for all analyzed groups. Regarding enteric exocrine drainage procedures, most UW, CS and HTK-preserved grafts were transplanted intraperitoneally (duodeno-jejunostomy), except for IGL-1, for which duodeno-duodenostomy technique was used in 69.6% of cases.

### Transplant Outcomes

#### Early Graft Function

**FIGURE 1:**
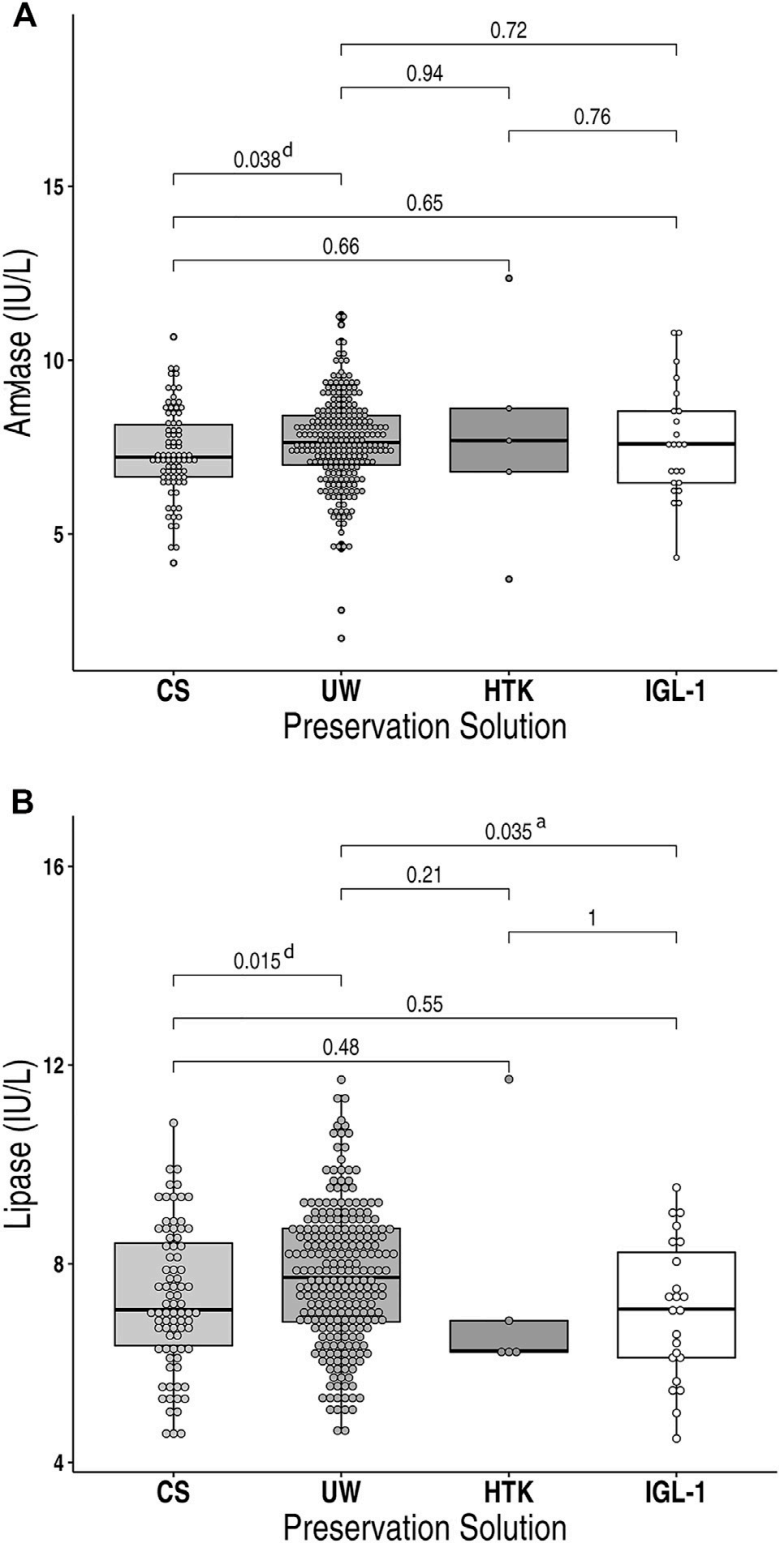
Box Plot showing **(A)** post-transplant serum amylase levels and **(B)** post-transplant serum lipase levels in recipients of pancreas allografts preserved in either CS, UW, HTK or IGL-1 preservation solution. Measured values were converted into logarithmic values. Boxes represent the interquartile ranges and whiskers extend to the minimum and maximum values. The median values are shown within the boxes. UW, University of Wisconsin; CS, Celsior; HTK, Histidine-Tryptophan-Ketoglutarate; IGL-1, Institut Georges Lopez-1. ^a^IGL-1 vs. UW; ^d^CS vs. UW.

On the immediate postoperative days (24h–48 h), serum amylase levels were the following: UW, 198 (IQR 127–341) IU/L; CS, 148 (IQR 100–295) IU/L; HTK, 206 (IQR 62–2821.5) IU/L and IGL-1, 193 (IQR 89–375) IU/L. Statistical differences were found between UW and CS (Figure 1A). The serum lipase levels were as follows: UW, 212 (IQR 114–420) IU/L; CS, 135 (IQR 80.5–350) IU/L; HTK, 76 (IQR 74.5–1738) IU/L and IGL-1, 136 (IQR 66–343.66) IU/L. The highest values for lipase peak were observed in the UW group, compared with CS and IGL-1 (Figure 1B). Despite the fact that those patients that required immediate transplantectomy were excluded from the amylase/lipase postoperative analysis, functioning pancreatic allografts perfused and subsequently preserved in HTK solution had an elevated serum amylase and lipase peak as demonstrated by IQR-75% compared to those preserved using other solutions. Nevertheless, the differences were not statistically significant.

Interestingly, a total of 30 patients presented kidney delayed graft function (DGF): UW (7.1%); CS (7.2%), HTK (57.1%); IGL-1 (4.3%), (*p* < 0.001, HTK vs. others). Hemodialysis was required in 15 of them in the immediate postoperative period, with progressive normalization of renal function at the moment of discharge.

#### Graft Transplantectomy

**FIGURE 2:**
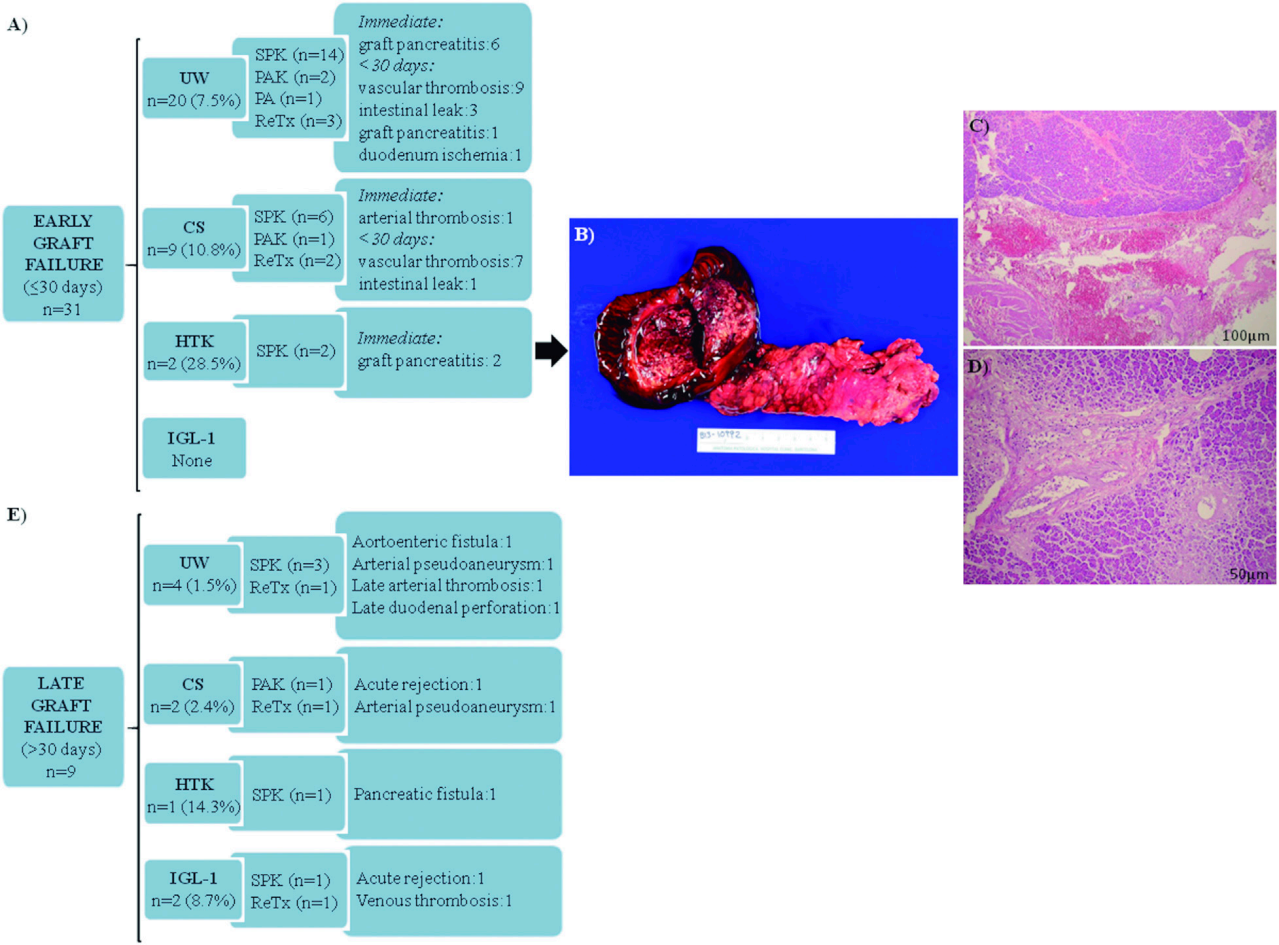
Pancreas graft failure requiring early (≤30 days) and late (>30 days) transplantectomy in transplanted patients. **(A)** Main causes of transplantectomy within 30 days after pancreas transplantation. Vascular thrombosis for UW group includes: venous thrombosis (*n* = 5); arterial thrombosis (*n* = 3); and venous + arterial thrombosis (*n* = 1). Vascular thrombosis for the CS group includes: venous thrombosis (*n* = 4); venous + arterial thrombosis (*n* = 3). **(B)** Macroscopic aspect of the graft perfused with HTK with areas of hemorrhage and necrosis in pancreatic tissue and duodenum. **(C)** Extensive hemorrhagic areas affecting the pancreatic parenchyma and peripancreatic soft tissue indicated by asterisks (H&E, scale bar 100 μm). **(D)** Pancreatic parenchyma with ischemic necrosis indicated by asterisks (H&E, scale bar 50 μm). **(E)** Main causes of transplantectomy after 30 days of pancreas transplantation. UW, University of Wisconsin; CS, Celsior; HTK, Histidine-Tryptophan-Ketoglutarate; IGL-1, Institut Georges Lopez-1; SPK, Simultaneous Pancreas-Kidney; PAK, Pancreas After Kidney; PA, Pancreas Transplant Alone; DM, Diabetes Mellitus; ReTx, Retransplant. H&E, Hematoxylin and eosin.

Early graft failure requiring transplantectomy within 30 days post-transplant occurred in 31 (8.1%) patients (Figure 2A), being more frequent in the case of HTK solution (28.5%). None of the IGL-1-preserved allografts required transplantectomy before 30 days. Vascular thrombosis was the main cause of early graft failure in UW and CS-preserved allografts, while graft pancreatitis was the leading cause of pancreatic failure in HTK-preserved allografts. Figure 2B illustrates the appearance of one of the HTK-perfused grafts, presenting an immediate severe macroscopic hemorrhagic reperfusion pancreatitis, and confirmed by histopathological data (Figures 2C,D).

Late causes of graft failure requiring pancreas transplantectomy beyond 30-day post-transplant accounted for 2.4% of the cases (Figure 2E).

#### Surgical Complications After Transplant

Technical failures in the total cohort amounted to 37.4% (*n* = 142). The Clavien-Dindo grading system for the classification of surgical complications was as follow: grade I (5.3%), grade II (6%), grade IIIa (3.9%), grade IIIb (20.5%), grade IVa (1.6%).

Focusing on the most relevant postoperative events, as depicted in Table 4, abdominal hemorrhage was identified in 8.4% of the cases (Grade A ISGPS (3.1%) and Grade B ISGPS (96.9%)), being similar between groups, except for IGL-1 group, which had none.

**TABLE 4:** Surgical postoperative complications.

	Total *n* = 380	UW *n* = 267	CS *n* = 83	HTK *n* = 7	IGL-1 *n* = 23	*P*
Pancreas						
Abdominal hemorrhage	32 (8.4)	24 (8.9)	7 (8.4)	1 (14.3)		0.133^a^
Clavien-Dindo						
I	1 (3.1)	1 (4.2)				0.150^b^
II	3 (9.4)	1 (4.2)	2 (28.6)			0.876^c^
IIIa						
IIIb	28 (87.5)	22 (91.6)	5 (71.4)	1 (100)		
IV						
Graft pancreatitis	14 (3.7)^*^	8 (3)	2 (2.4)	3 (43)	1 (4.3)	
Clavien-Dindo						
I	1 (7.1)				1 (100)	0.720^a^
II						0.620^b^
IIIa	5 (35.7)	2 (25)	2 (100)	1 (100)		0.779^c^
IIIb						
IV						
Abdominal fluid collection	10 (2.6)	7 (2.6)	3 (3.6)			
Clavien-Dindo						
I	2 (20)	1 (14.3)	1 (33.3)			0.432^a^
II	2 (20)	2 (28.6)				0.355^b^
IIIa	1 (10)		1 (33.3)			0.635^c^
IIIb	5 (50)	4 (57.1)	1 (33.3)			
IV						
Intestinal complication	25 (6.6)	15 (5.6)	8 (9.6)	1 (14.3)	1 (4.3)	
Clavien-Dindo						
I	3 (12)	1 (6.7)	2 (25)			0.798^a^
II	2 (8)		1 (12.5)	1 (100)		0.421^b^
IIIa						0.197^c^
IIIb	14 (56)	10 (66.7)	3 (37.5)		1 (100)	
IV	6 (24)	4 (26–7)	2 (25)			
Vascular thrombosis^**^	78 (20.5)	57 (21.3)	17 (20.5)		4 (17.4)	0.655^a^
Anticoagulation protocol	23 (29.5)	20 (35.1)	3 (17.6)			0.742^b^
Conservative	11 (14.1)	5 (8.8)	4 (23.5)		2 (50%)	0.866^c^
anticoagulation	19 (24.4)	17 (29.8)	2 (11.8)			
Interventional radiology	25 (32.1)	15 (26.3)	8 (47.1)		2 (50%)	
Relaparotomy						
Pancreas graft (nº patients)	83 (21.8)	58 (21.7)	21 (25.3)	1 (14.3)	3 (13)	0.327^a^
Time after transplant (days)	6 (2–15)	6.5 (1.7–15)	4 (1–12.5)	2	19 (3–36)	0.214^b^
—						0.496^c^
Hospital stay	15 (11–22)	14 (11–21)	15 (12–24)	30 (11–34)	13 (11–19)	0.475^a^
						0.257^b^
						0.384^c^

Categorical variables are expressed as frequencies (%) and percentages and continuous variables as median and interquartile range (IQR).

Comparison of the analysed variables have been made between UW, CS, and IGL-1, groups. For HTK, group only a descriptive analysis is displayed.

a IGL-1, vs. UW

b IGL-1, vs. CS

c UW, vs. CS.

Include hemoperitoneum, intra-abdominal/subcutaneous hematoma.

^*^In 8 of the cases an immediate transplantectomy was required, not included in Clavien-Dindo classification.

^**^Venous and arterial thrombosis.

Anticoagulation protocol (enoxaparin + aspirin).

Conservative Anticoagulation (systemic heparin/acenocoumarol).

UW, indicates University of Wisconsin; CS, Celsior; HTK, Histidine-Tryptophan-Ketoglutarate; IGL-1, Institut Georges Lopez-1.

In most cases, a surgical reintervention was required due to: hemoperitoneum (*n* = 24); intra-abdominal hematoma (*n* = 3); and subcutaneous hemorrhage (*n* = 1). Graft pancreatitis was diagnosed in a total of 14 patients. There were numerical differences based on the preservation solution type (*p* < 0.001), with a significantly high rate for the HTK group (43%, thymoglobulin (*n* = 2), basiliximab (*n* = 1)). Regarding the whole series, some 8 cases required an immediate transplantectomy because of a severe graft necro-hemorrhagic pancreatitis after reperfusion (Figures 2A–D). In those situations, the surgeon considered the graft not viable after checking the tightness and absence of thrombi of the vascular anastomoses. Another HTK case presented a less severe heterogeneous reperfusion with areas of intra-parenchymal hemorrhage (amylase/lipase at 24 h: 5250/3369 IU/L). In that situation, it was decided to salvage the graft, although a pancreas transplantectomy was mandatory 7 months later because of an infected persistent pancreatic fistula. The remaining 5 cases of pancreatitis, presented with a median 24 h serum values of amylase and lipase of 1017 IU/L (796.25–2007) and 776.5 IU/L (495.1–1968.7) respectively, evolved as peripancreatic fluid collection, requiring relaparotomy (*n* = 4) at a median of 12 days (6.5–15) post-transplant and percutaneous abscess drainage (*n* = 1). Other intra-abdominal fluid collections were diagnosed in 10 patients, without impact on graft survival. A relaparotomy was needed in half of them, performed in most cases when the patient was readmitted after discharge because of fever and abdominal pain at a median of 28 days (18–43.5) after transplant. Intestinal complications (6.6%) included post-transplant duodenal-enteric leaks and those related to small-bowel obstruction. A total of 5 patients required early transplantectomy because of: anastomotic leak (*n* = 2); a leak of the duodenal stump site (*n* = 2), and ischemia of the duodenum (*n* = 1). The UW, CS and IGL-1 preservation solutions presented similar rates of vascular thrombosis (venous (77%), arterial (6.4%), both (16.6%). Note that, in a total of 25 out of 78 patients, surgery was applied as treatment. Other therapeutic options used for thrombosed pancreas grafts are also described in Table 4.

#### Patient and Graft Survival

**FIGURE 3:**
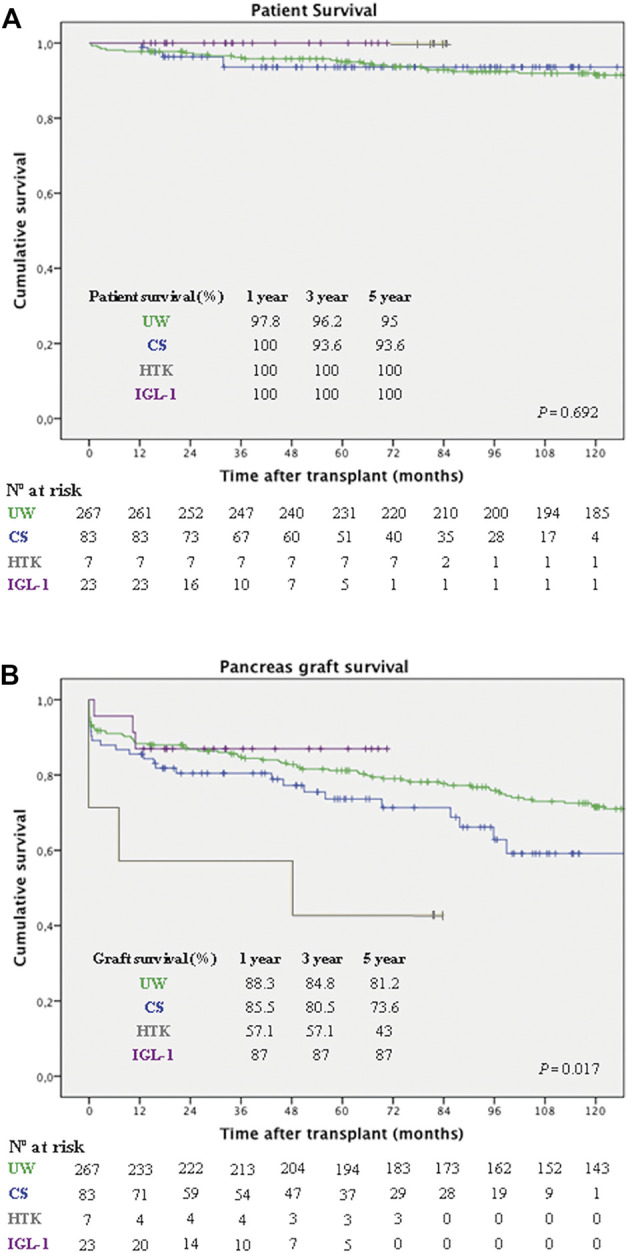
Pancreas graft and patient survival in 380 consecutive pancreas transplants according to preservation solution. **(A)** Patient survival rates at 1, 3, and 5 years were: 97.8%, 96.2%, and 95%, respectively for the UW group (green line); 100%, 93.6%, and 93.6%, respectively for the CS group (blue line); 100% for the HTK group (grey line) and 100% for the IGL-1 group (purple line), with no significant differences (*p* = 0.692, LR). **(B)** Death-censored pancreas graft survival rates at 1, 3, and 5 years were: 88.3%, 84.8%, and 81.2%, respectively for the UW group (green line); 85.5%, 80.5%, and 73.6%, respectively for the CS group (blue line); 57.1%, 57.1%, and 43%, respectively for the HTK (grey line) group; and 87%, 87%, and 87%, respectively in the IGL-1 group (purple line) (*p* = 0.017, LR). UW, University of Wisconsin; CS, Celsior; HTK, Histidine-Tryptophan-Ketoglutarate; IGL-1, Institut Georges Lopez-1; LR, Log-rank test.

After a median follow-up of 118.4 months (IQR: 63.2–168.9), overall patient survival for the whole cohort at 1, 3, and 5 years was 98.4%, 96%, and 95%, respectively. Patient survival rates at 1, 3, and 5 years for the studied groups are depicted in Figure 3A, with no significant differences between them (*p* = 0.692).

Overall death-censored pancreas graft survival for the whole cohort at 1, 3, and 5 years was 87.1%, 83.4%, and 79%, respectively. Figure 3B represents the pancreas graft survival rates at 1, 3, and 5 years for the different preservation solution groups. Overall UW, IGL-1 and CS were associated with better pancreas graft survival, compared to HTK (*p* = 0.017).

Regarding pre-procedure variables related to donor and recipient, a significantly increased risk of graft loss on univariate analysis was associated with the following: CIT (>10 h), [hazard ratio (HR) 1.51, 95% CI 1.02–2.23; *p* = 0.035], HTK as preservation solution (HR 3.48, 95% CI 1.27–9.52; *p* = 0.009), pretransplant creatinine (>5.9 mg/dl) (HR 0.66, 95% CI 0.44–0.98; *p* = 0.039), type of transplant (other than SPK) (HR 2.12, 95% CI 1.38–3.25; *p* < 0.001), recipient gender (female) (HR 1.52, 95% CI 1.03–2.23; *p* = 0.031). Other variables with no statistical significance yet presented a tendency to influence graft survival were: donor BMI >27 kg/m^2^ (*p* = 0.057) and donor cause death other than trauma (*p* = 0.06). In a multivariate Cox regression model for graft survival, the variables associated with an increased risk for graft failure were: type of transplant (other than SPK) (HR 5.46 CI 1.63–18.28; *p* = 0.005) and recipient gender (female) (HR 1.97, 95% CI 1.00–3.86; *p* = 0.04).

## Discussion

Of all solid organ transplant types, pancreas transplants are most susceptible to non-immunologic failure, with a reported graft loss rate of 5%–20% during the first year after transplantation (18-20). Because of the high vulnerability of the pancreas, an appropriate preservation solution could make a difference on graft and patient outcome. However, there is no universal consensus concerning the optimal preservation fluid in PTx (12).

Herein, we present the first retrospective single-center study comparing the effects of the four most commonly used preservation solutions in PTx, i.e. UW, CS, HTK, and IGL-1, on early pancreatic graft function as well as long-term patient and graft survival. By analyzing a large cohort of pancreas transplants in a 20–year period, this study shows that, although similar rates of graft survival were observed during the first year when comparing IGL-1, CS and UW, better results for IGL-1 were observed over the long term. Conversely, the HTK-preserved pancreas had the lowest graft survival in comparison to the other preservation solutions employed, supporting the findings of Hameed AM et al. (12) when comparing UW, HTK and CS preservation solution in a meta-analysis study.

Of note, out of the total 31 cases with early graft failure requiring transplantectomy within 30 days post-transplant, none were associated with the use of IGL-1 preservation solution. However, even though this result seems promising, they need to be interpreted cautiously because of the small sample size of IGL-1 cohort in comparison with UW or CS. When analyzing the intraoperative events, severe reperfusion pancreatitis with immediate graft removal was present in 28.5% of preserved-graft with HTK, a higher percentage when compared to other solutions. Clinical experience with HTK solution still generates controversy. It is known that its low viscosity necessitates larger solution volumes, as initially recommended by the manufacturers. However, it has been demonstrated that this factor may also be detrimental for optimal pancreas preservation, and that abdominal organs can be adequately preserved with a total volume of 5–7 L of HTK (21). In the majority of clinical studies, the HTK-flushed grafts had a higher risk of graft loss due to acute pancreatitis and thrombosis when experiencing ischemic times in excess of 12 h (22-24). In our cohort, the median of HTK-perfused solution used was 7 L. Despite the fact that HTK was used in grafts with shorter CIT, and that no changes were made in organ recovery practices, transplant techniques, or transplanting surgeons, a significant increase in the rate of pancreatitis in recipients was observed (*p* < 0.001). These findings are in contrast to a larger series published by Fridell et al. (25), who found no differences in outcomes of 308 pancreas transplants with the use of UW and HTK, suggesting that the observed differences in other studies may have been attributed to long ischemic times (19) and larger flush volumes.

A study from Ngheim et al. suggested that dual perfusion may alter pancreatic function during pancreas procurement in comparison to the aortic-only vascular perfusion (26). The authors found that the 6 pancreas retrieved by dual aortic and portal flush had higher serum amylase and lipase levels and lower levels of urine bicarbonate and pH. However, due to the lack of larger studies, both single and dual perfusion are currently considered as effective methods when procuring the pancreas for transplantation (12, 27). The impact of this factor could not be evaluated in the present series as aortic-only perfusion was not investigated. However, this method could be a source of future research to assess whether or not dual perfusion is a possible risk factor for increased graft injury resulting from venous congestion and graft edema.

Although vascular thrombosis has been shown to be a risk factor for graft loss (28-34), in this series no differences have been observed in relation to the preservation solution used. The same applies to intestinal-related morbidity.

Another important consideration when analyzing the results of our series is the quality of the pancreatic donor. Examination of the records showed no statistically significant differences regarding donor characteristics and preservation solutions used, with the exception of older pancreatic grafts in the HTK and CS groups, and longer CIT for UW and CS cases. Studied groups were also similar regarding recipient characteristics, with the exception of older patients for IGL-1 group, and longer DM *vintage* for HTK group.

No active interventions among pre-procedure factors with influence on graft survival, such as the recipient gender or type of transplant, are possible as they are unchangeable variables. Moreover, and taking into account the heterogeneous population and the long-time study period, neither the era of study (before and after 2010, as it was the midpoint of the period (2000–2019)), the type of vascular reconstruction nor the intestinal anastomosis had an impact on the early graft functioning.

In general, our findings are consistent with the scant published information in PTx using IGL-1. At the clinical level, one preliminary study suggests that IGL-1 is a safe preservation solution since it provides up to 17 h of cold ischemia. The five human pancreases preserved with IGL-1 acquired normal function immediately after reperfusion, without loss of the graft (35). Similar results were observed in a more recent study comprising a series of 47 consecutive PTx (36). Conversely, IGL-1 has been proven to be equivalent to UW or CS solutions for pancreas perfusion and cold storage before islet transplantation (37). Nevertheless, in a model of PTx in pigs, IGL-1 offered greater protection in membrane fluidity after reperfusion (38).

To the best of our knowledge, this is the only study exploring the effect of the four preservation solutions currently used for clinical PTx. We are aware that the suboptimal number of patients (mainly in the HTK group) limit the conclusions of the study, even though this factor is mitigated when evaluating the results from the point of view of “intention to treat”. A low number of HTK-flushed pancreases has arisen due to an unexpected increase in the rate of immediate transplantectomy due to acute pancreatitis following reperfusion, as the latter is also an independent risk factor for impaired graft survival. This fact limited HTK’s use in PTx and did not allow us to recruit an optimal number of cases for comparison with a suitable sample size. Furthermore, no hard conclusion could be obtained on the influence of induction therapy on technical failure as two out of the three cases with adverse effect were treated with thymoglobulin, which has potential broad anti-inflammatory properties that have been shown to reduce ischemia-reperfusion injury (39, 40). A long period time study carries with it inherent improvements in perioperative patient care, surgical technique and postoperative management, but the present series transplant era in question did not have statistically significant influence on the graft outcomes. Finally, the fact that surgical technique was changed in 2016 to duodenoduodenostomy does not affect immediate reperfusion injury rates, as vascular anastomoses were performed with the same technique throughout the time period in question. Despite numerous techniques to minimize exocrine pancreatic drainage complications, no universal technique has been standardized (41,42). To date, it is unclear whether duodenojejunostomy or duodenoduodenostomy provides the best long-term survival of the grafts (43). A prospective multicentre registry analysis may resolve this.

In conclusion, the fruits of this study indicate a trend towards a better graft and patient survival among IGL-1 recipients. Besides, IGL-1 composition is similar to that of the UW solution, currently considered as the “gold standard” in the reduction ischemia-reperfusion injury of the pancreas. Hence, successful PTx can be safely performed using IGL-1 solution. Further multicenter studies are still required to identify the “holy grail” of preservation solutions, especially in the current scenario of using marginal donors, including donors following circulatory death, in which the graft is exposed to a warm ischemia insult before cold storage, raising susceptibility to graft dysfunction.

## Data Availability

The original contributions presented in the study are included in the article/Supplementary Material, further inquiries can be directed to the corresponding author.

## Abbreviations

BMI, Body Mass Index; CIT, Cold Ischemia Time; CS Celsior; DGF, Delayed Graft Function; DM, Diabetes Mellitus; HTK, Histidine-Tryptophan-Ketoglutarate; HR, Hazard Ratio; IGL-1, Institut Georges Lopez-1; IQR, Interquartile Range; ISGPS, International Study Group for Pancreatic Surgery; LR, Log-rank Test; PAK, Pancreas After Kidney; PPASS, Pre-Procurement Pancreas Suitability Score; PTA, Pancreas Transplant Alone; PTx, Pancreas transplantation; SPK, Simultaneous Pancreas-Kidney; UW, University of Wisconsin.
